# Effects of lay support for pregnant women with social risk factors on infant development and maternal psychological health at 12 months postpartum

**DOI:** 10.1371/journal.pone.0182544

**Published:** 2017-08-28

**Authors:** Emma Popo, Sara Kenyon, Sophie-Anna Dann, Christine MacArthur, Jacqueline Blissett

**Affiliations:** 1 Institute of Applied Health Research, College of Medical and Dental Sciences, University of Birmingham, Edgbaston, Birmingham, United Kingdom; 2 Centre for Technology Enabled Health Research, Coventry University, Coventry, United Kingdom; 3 School of Psychology, University of Birmingham, Edgbaston, Birmingham, United Kingdom; TNO, NETHERLANDS

## Abstract

**Background:**

The ELSIPS (Evaluation of Lay Support in Pregnant Women with Social Risk) RCT showed that lay support for women with social risk had a positive effect on maternal mental health and mother-infant bonding. This exploratory study examined whether these observed benefits would impact infant development at 1 year.

**Methods:**

A sub-sample of women whose infants were under one year who had participated in the ELSIPS RCT which randomised women to receive either standard care or the services of a Pregnancy Outreach Worker (POW), and who were contactable, were eligible to participate in the follow up. At home visits, the Bayley Scales of Infant Development (3^rd^ Edition) and standardised measures of depression, self efficacy, mind-mindedness and bonding were completed.

**Results:**

486 women were eligible for follow up, of whom 154 agreed to participate. 61/273 were successfully followed up in the standard maternity care arm and 51/213 in the POW arm. Women who completed follow up were less depressed and had higher selfefficacy scores at 8–12 weeks postpartum than those who did not complete follow up. There were no significant differences in maternal outcomes, infant cognitive development, receptive communication, expressive communication, fine motor development or social/emotional functioning between groups at 12 month follow up. Infants of mothers who received the POW intervention had significantly better gross motor development than infants whose mothers received standard care (p<0.03).

**Conclusions:**

The provision of lay support to women with social risk may facilitate infant gross motor skill development at one year but there were no other demonstrable benefits. The effects of the intervention may be underestimated given that those women who completed follow up had better mental health than the original study sample.

**Trial registration:**

Controlled-Trials.com ISRCTN35027323.

## Introduction

Maternal and child health outcomes for disadvantaged and minority families continue to fall behind those with more advantaged backgrounds [1,2,3]. Severe maternal morbidities are more likely in non-white ethnic groups, with preexisting health conditions and poorer access to health services being risk factors for higher morbidity [4]. Women with complex social risk factors such as domestic violence and substance abuse are more likely to seek antenatal care later and fail to stay in regular contact with health services during pregnancy [4]. The differences in health behaviors and outcomes between socially advantaged and disadvantaged women and children are mediated by a number of factors including lack of social support [5,6], lack of education [7,8] and poor living environment [9].

Inequalities in health are not limited to physical functioning; for example women with higher socioeconomic status have lower rates of depression [10]. Postpartum depression (PPD) is characterized by low maternal mood within the year after childbirth [11] and is associated with reduced sensitivity in interaction and emotional withdrawal [12,13]. The effects on child development include adverse child physical, social and emotional developmental outcomes such as poor cognitive development and greater likelihood of insecure attachment [13–17]. Depressed women are less likely to develop warm, loving feelings towards their children [18–20] and failure to bond has implications for the subsequent relationship. Disordered bonding is characterized by a significant lack of maternal feeling; hostility toward the infant, lack of responsiveness to the infant’s needs, and sometimes, harm or neglect [21,22]. Depression is also associated with reduced maternal ‘mentalising’ and vocalizations about infant emotion and cognition [23]. This characteristic is called ‘mind-mindedness’; the mother’s tendency to view her child as an individual with a mind of its own [24,25]. There is significant individual variation in mothers’ mind-mindedness, and poorer mind-mindedness is associated with poorer infant cognitive development [26] and less secure attachment [27,28]. Mind-mindedness is also associated with parenting stress and child emotional and behavioural problems and has been highlighted as an important target for interventions [29]. The ability to appropriately comment on infant mental states and processes facilitates infant cognitive and socioemotional development via increased maternal sensitive responsiveness [30].

Another important predictor of maternal functioning is self efficacy; the extent to which individuals regard their lives as under their own control [31]. Depression is associated with reduced perceptions of personal control [32]. Self efficacy plays a crucial role in parenting behaviour and infant psychosocial risk [33, 34]. Self efficacy is a primary predictor of parenting behavioural competence in mothers of young infants, over and above the effects of depression and social support [35]. Given that perceived control over life events has been suggested to underlie social inequalities in health, interventions that seek to increase self efficacy have the potential to improve social, physical and mental health outcomes for mothers and their infants.

Research has begun to assess the efficacy of a variety of types of support for women with high social risk during and after pregnancy, including lay support. Lay support is provided by community workers or peers who are not health professionals but whose role is to provide social and practical support, for example, to access existing care pathways. A Cochrane review of psychosocial and psychological interventions for preventing postpartum depression included seven lay interventions, and demonstrated that they were not significantly different from professionally delivered interventions in their ability to reduce risk of depression [36]. However, the review called for further trials of the efficacy of lay support because of the small number of eligible studies, the lack of trials examining individual lay interventions targeting mood, and the lack of detail concerning the nature of the lay individuals delivering the intervention [36]. The Evaluation of Lay Support in Pregnant Women with Social Risk (ELSIPS) study [37] aimed to evaluate whether using lay Pregnancy Outreach workers (POW), could benefit maternal and infant health outcomes in nulliparous women with a number of social risk factors. POWs were trained by an independent Community Interest Company to deliver individual case management for women identified by the midwifery team as having social risk. The service included home visits, and aimed to encourage antenatal attendance and healthy lifestyles and facilitate access to existing services (e.g. benefits, housing). The POW’s role was also to provide support to mothers to improve their psychological health, including depression, self efficacy and bonding. Most contacts between POWs and the women in the trial took place antenatally (77%), with 27% being face to face contact and half of contacts lasting between 1–2 hours (see http://dx.doi.org/10.1136/bmjopen-2015-009203 [38] for full trial details). The trial found modest positive effects on maternal mental health at 8–12 weeks postpartum, particularly in those women with greater social risk [38] and a significant benefit for mother-to-infant bonding. Measuring the potential impact of these effects on infant development is important given the cumulative duration and timing effects of maternal depression on infant outcomes [39].

This study aimed to follow up a subsample of families within the ELSIPS trial to evaluate infant and maternal outcomes at one year postpartum. The aims were 1) to examine whether the service had a positive impact on the cognitive, social, emotional and physical development of the infant at 12 months and 2) to examine whether the POW service had an effect on maternal depression, mind-mindedness, self efficacy and bonding at 12 months postpartum.

## Materials and methods

ELSIPS was a randomised controlled trial (RCT) in nulliparous women with social risk, which compared standard maternity care with the addition of referral to a lay Pregnancy Outreach Worker (POW) support service. The POWs worked alongside community midwives offering individualised support to encourage engagement with health and social care services, from randomisation (before 28 weeks gestation) until 6 weeks after birth. Primary outcomes were engagement with antenatal care and maternal depression at 8–12 postnatal weeks, using the Edinburgh Postnatal Depression Scale (EPDS [40]). Self efficacy and mother-to-infant bonding was also measured at 8–12 weeks. Details of the original ELSIPS trial have been previously published [37, 38]. 1324 women participated in the original trial, 662 receiving the intervention and 662 standard care.

### Study oversight

Ethical approval from South Birmingham Ethics Committee (10/H1207/23). Participants in the original trial received a separate information leaflet for the follow up study and signed a consent form specific to the follow up study before participation.

### Follow up study design

We followed up a subsample of women and infants in both trial arms to evaluate the effect of the intervention at 12 months postpartum on the primary outcome of infant development (measured by the Bayley Scale of Infant and Toddler Development-III [41]). Selection criteria were applied systematically and researchers were blind to participant group membership at the point of decisions regarding eligibility for follow up.

The secondary outcomes for the follow up were maternal depressed mood, mind-mindedness, self efficacy and maternal bonding. Infant weight and length were also recorded.

The researcher conducting home visits and collecting and entering all data was blind to group membership and maternal social risk factors.

### Participants

**Fig 1:**
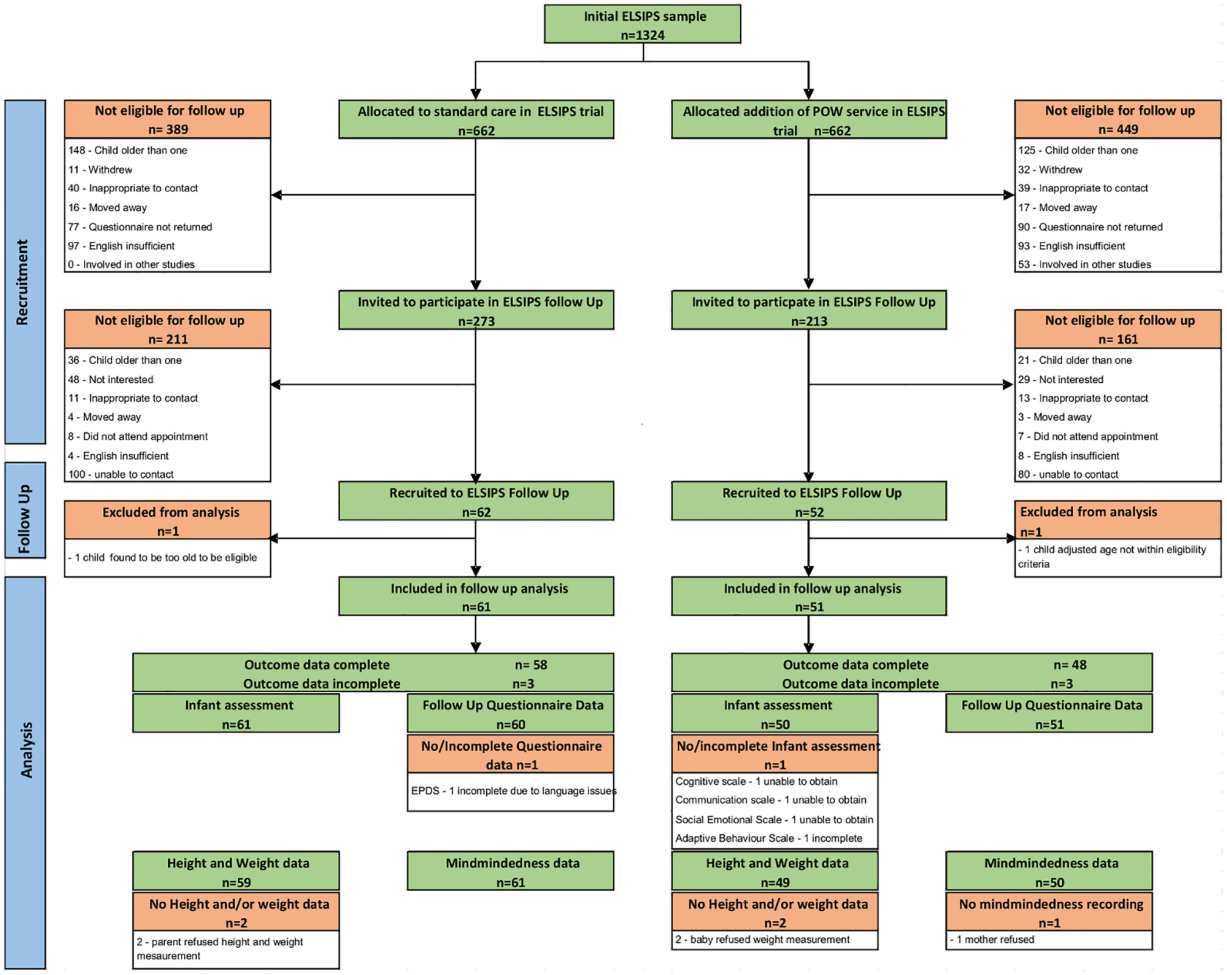
Consort diagram for the ELSIPS follow up.

We followed up women whose infants were between 11–13 months between June 2012 and June 2013. This was a pragmatic sample; participants who could be included were limited due to funding and time constraints. Women were eligible if they had returned an 8–12 week questionnaire in the original trial, and gave birth to a live infant after 1^st^ June 2011. Women were not eligible if they had been approached to engage in other additional research or evaluations of the trial (e.g. qualitative analyses), if we did not have accurate contact information, if they did not speak English, or if the GP felt that participation was inappropriate. Of the original sample of 1324 mother and baby pairs, this left a potential pool of 486 participants. See Fig 1.

When infants were aged 7 to 11 months, their mothers were invited to participate by sending a patient information leaflet and reply slip to their home. Once the reply slip was returned to the study office, the mother was telephoned to confirm participation, or the mothers were called directly by the researcher when their child approached 11 months old to book a home visit and check eligibility.

155 mothers were engaged in total, representing 11.7% of the original trial sample. In total, 8.5% of the original sample were successfully followed up. Home visits could not be completed in 43 cases, due to the mother not being present at the agreed appointment (n = 41) or the child exceeding the age cut off by the time of booking (n = 2). If a mother did not attend an arranged appointment, two further attempts were made to contact her to re-arrange. If a mother did not attend a second appointment, a final attempt was made to re-arrange the appointment but no further contact was made after this point.

### Measures

#### Primary outcome: Infant development

The Bayley III Scale of Infant and Toddler Development (Bayley-III) measures the mental, motor and behavioural development of infants from one to 42 months of age across 5 subscales: cognitive, language, motor, social/ emotional and adaptive behaviour [38]. It takes 45–60 minutes to complete. The Bayley Scale is a widely used, internationally recognised, extensively evaluated, standardised, reliable and valid gold standard for early childhood assessment [42].

The cognitive, language and motor scales are administered by the researcher (in this case a psychologist) who has attended accredited training. The Cognitive Scale assesses the infant’s information processing and play skills. The Language Scale is composed of receptive communication (infant’s preverbal behaviour, social referencing and verbal comprehension) and expressive communication (infant’s preverbal communication skills, including gestures). The Motor Scale is split into fine motor (ability to manipulate objects, reaching, grasping and visual tracking) and gross motor (how well the child can move his /her torso and limbs, balance and static positioning).

The social/emotional and adaptive behaviour questionnaires are completed by the caregiver. The Social Emotional Scale assesses the social and emotional functioning of the child reported by the caregiver, identifying whether key social-emotional milestones have been reached. The Adaptive Behaviour Scale assesses the child’s skills in behavioural autonomy and responses to his/her environmental context.

#### Secondary outcomes: Maternal cognition and emotion

Maternal mind-mindedness can be measured by the mother’s use of mental state language in descriptions of her child [26]. All mothers were asked to ‘describe (your baby’s name) for me’, and responses were audio-recorded, transcribed and coded by two trained independent coders (both psychologists, both trained in mind-mindedness coding) blinded to group allocation and social risks. The mind-mindedness interview has established reliability, validity and feasibility [43].

Mothers completed three questionnaire measures, which had also been completed at 8–12 weeks postpartum. These were:

Edinburgh Postnatal Depression Scale [40]: A 10 item self report questionnaire with 4 response options, designed to assess emotional wellbeing. A score of 13 or higher suggests depression. The EPDS is the most frequently used measure of postnatal depression, with good reliability, validity, specificity and sensitivity [44].
Pearlin Mastery Scale [31]: A 7 item index of self efficacy, with 4 point likert response options (strongly agree to strongly disagree). Higher scores indicate higher self efficacy. This scale has been used in many large scale studies to examine parents’ self efficacy [45] (for example, the National Survey of Families and Households, as well as the National Longitudinal Survey of Youth).
Mother to Infant Bonding Scale [46]: Seven expressions indicating the mothers feelings toward her child, followed by a 4 point likert scale (Very much to Not at all). Higher scores indicate poorer maternal to infant bonding. The scale is widely used with good reliability and validity [47].
Length and weight: Infant length and weight was measured using the SECA 210 Measure Mat and the SECA 384 electronic scale.

### Procedure

After informed consent, the mind-mindedness interview was conducted. Next, self-report measures were completed. The infant’s height and weight was measured by the researcher, fully clothed for height measurement but clothes and nappy removed for weight measurement.

The Bayley assessment was administered in the following order: Gross motor, fine motor, cognitive, receptive communication and expressive communication. The child’s age was calculated and for premature babies, the adjusted age was calculated using gestation at birth. Standard administration procedure was followed. On completion of the assessment and questionnaire, the mother was given £20 in high street vouchers. The same researcher conducted all measures in both arms of the trial.

### Data analysis

#### Bayley scale scores

Raw scores were summed for each subtest and converted into scaled scores. Scaled scores were used to determine the child’s performance against norms from typically developing children (reference mean = 10, SD = 3).

#### Mind-mindedness

After transcription, each comment was coded as mind-minded (referring to a mental state), general, behavioural or physical. The percentage of mind-related comments relative to the total number of comments was calculated. Inter-rater reliability was calculated; 90.5% of the statements were coded in the same way by the two coders, suggesting overall agreement was very good (Kappa = .86).

We examined differences in social risk and other characteristics between those participants who did and did not complete the follow up study irrespective of eligibility for follow-up, using independent sample t-tests. We also examined differences between those participants who were eligible for the follow-up and who did or did not complete the study, using independent sample t-tests. Differences between arms in outcomes were compared using ANOVA where data were normally distributed and Mann Whitney U tests where the data were significantly skewed. Differences between groups in the percentage of participants who had an EPDS score ≥13 were analysed using chi-square. Analyses of differences in self efficacy, depression and bonding at 1 year follow up were adjusted for a group difference in gestational age (see below) with ANCOVA. Analysis of infant developmental outcomes was not adjusted for gestation because the measures used to assess infant development adjust for gestational age. Based on the sample available post hoc power analyses showed that with 112 participants and alpha at .05, power of .95 was available to detect effect sizes of .38 and larger; this corresponds to a difference between arms of 1.14 Bayley scale points (mean score for Bayley scaled subscales is 10 and standard deviation is 3). We also carried out pre-specified subgroup analyses of the women who had 2 or more social risks at baseline.

SPSS software (IBM SPSS statistics version 21) was used for statistical analyses.

## Results

### Sample characteristics

Table 1 illustrates characteristics of participants followed and not followed from the original trial. Of the 112 mothers visited, 62 infants were boys and 50 were girls. The follow up sample was largely representative of the initial and eligible ELSIPS sample though there were proportionately fewer Asian mothers in the follow up, due to the eligibility criteria of good understanding of written and spoken English (46.6% of ineligible Asian mothers did not speak English). There were no significant differences in social risk count, birth weight or gestation between those who did and did not participate in follow up, irrespective of eligibility. However, there was a significant difference in EPDS and self efficacy: women who participated in follow up had significantly lower mean depression scores (t = 2.94, df = 1006, p = .003) and reported greater self efficacy (t = -3.11, df = 975, p = .002) than those who did not participate overall, and than those who were eligible but did not participate (depression t = 2.7, df = 217, p = .007; self efficacy t = -2.8, df = 220, p = .005). There was no significant difference between those followed up and those not followed, irrespective of eligibility for follow up, in proportions of EPDS ≥13.

**Table 1:** Baseline data for ELSIPS follow up sample compared to a) all participants who were eligible to be followed but who were not followed and b) all participants who were not followed.

N = 1324	Followed Up	Eligible for follow up but not followed	Not Followed Up (overall sample)
Group total (%)	112 (8.5)	373 (28)	1212 (91.5)
Ethnicity			
White European (%)	80 (71.4)	241 (65)	609 (50.2)
African (%)	5 (4.5)	14 (3.7)	82 (6.8)
Caribbean (%)	12 (10.7)	20 (5.3)	58 (4.8)
Asian (%)	6 (5.4)	69 (18.4)	335 (27.6)
Other (%)	9 (8.0)	24 (6.4)	86 (7.1)
Middle Eastern (%)	0 (0.00)	5 (1.3)	42 (3.47)
Mean birth weight (sd)	3152 (632)	3199 (563)	3167.8 (617.3)
Mean Gestation in days (sd)	-2.5 (13.1)	-2.3 (14)	-3.6 (16.5)
Mean baseline EPDS score (sd)	5.7 (4.5)*	7.1 (5.4)	7.2 (5.4)
N(%) ≥13	10 (8.9)	54 (11.1)	138 (11.4%)
Mean baseline Self efficacy score (sd)	20.0 (2.7)*	19.2 (3.2)	19.0 (3.2)
Mean baseline MIB score (sd)	1.9 (.2)	1.9 (.2)	1.9 (.2)
Social risk count (sd)	2.3(1.0)	2.2(1.1)	2.2(1.1)

*Followed up sample are significantly different (*p* ≤ 0.05) from overall ELSIPS sample not followed AND significantly different from sample eligible for follow up but not followed at 8–12 weeks postpartum.

Of the 112 women and infants included, 51 were from the POW service and 61 from the standard care trial arms. Table 2 shows the descriptive statistics at 8–12 weeks for the participants followed up in each arm. When comparing the sample by randomisation group, there were no significant differences in demographic and anthropometric characteristics, except that infants in the POW group had significantly fewer days of gestation (t = 2.0, df = 111, p = .05). Analyses of maternal outcomes were therefore adjusted for gestation. There were small differences in maternal mental health between the POW and control arms at 8–12 weeks postnatal, with a trend towards a lower mean levels of depression (t = 1.7, df = 110, p = .09) and significantly higher self efficacy scores (t = -2.2, df = 109, p = .03) in the women who had received the POW intervention. There were no significant differences between the POW group and standard care group in the percentage of participants with EPDS scores ≥13.

**Table 2:** ELSIPS sollow up sample composition and characteristics.

	Trial Group		
	Standard	POW	total
ELSIPS sample			1324
Eligible to participate			486
Recruited (%)	61 (54.5)	51 (45.5)	112
Boy (%)	33 (55)	29 (55.8)	
Girl (%)	27 (45)	23 (44.2)	
Ethnicity			
White European (%)	46 (46.7)	34 (65.1)	
African (%)	1 (1.7)	4 (7.7)	
Caribbean (%)	5 (8.3)	7 (13.5)	
Asian (%)	4 (6.7)	2 (3.8)	
Other (%)	4 (6.7)	5 (9.6)	
Birthweight g (sd)	3199.5 (603.6)	3096.2 (665.7)	
Male Infant weight in kg at 12 months (sd)	10.3 (1.4)	10.1 (1.1)	
Female Infant weight in kg at 12 months (sd)	9.4 (1.3)	9.5 (1.4)	
Male Infant length in cm at 12 months (sd)	76.5 (2.9)	76.4 (2.3)	
Female Infant length in cm at 12 months (sd)	74.8 (2.6)	74.9 (3.9)	
Gestation in days (sd)	-0.5 (10.2)	-4.9 (15.5)*	
Social risk count (sd)	2.2 (1.1)	2.5 (0.9)	
Baseline EPDS mean (SD)	6.3 (4.6)	4.9 (4.2)^t^	
N (%) ≥13	7(11.4)	3 (5.8)	
Baseline Mastery (self efficacy) mean (SD)	19.5 (2.8)	20.6 (2.4)*	
Baseline Bonding mean (SD)	22.9 (1.5)	23.0 (1.2)	

^t^ p < .1

*p ≤ 0.05 Baseline measures taken at 8–12 weeks postpartum.

### Inferential analysis

There were no differences between intervention and control arms of the trial in maternal depression, bonding or self efficacy outcomes at 12 months postpartum (Table 3). Despite this, infants of the women who had received the POW intervention had significantly better gross motor skills than those who received standard care, with the infants in the POW arm scoring close to the scaled reference mean of 10. There were no other significant differences between the two groups of infants in developmental outcome. Scores of cognitive development and receptive communication were approximately one standard deviation lower than the scaled reference indicating below average performance of infants in both the POW and Control trial arms. Sub group analyses of women with 2 or more social risks showed that there were no significant differences in infant development between infants of women with 2 or more social risks in the POW (n = 45) and control (n = 43) arms of the trial, and no differences in the two groups of women in their longer term depression, self efficacy or bonding scores (see S1 and S2 Tables).

**Table 3:** Group differences by randomisation in the ELSIPS follow up study at 12 months postpartum.

Outcome variable	Treatment Group			AN(C)OVA/chi square
	Standard (n = 61)	POW (n = 51)	N	F/X2, *p-value*
**Primary Outcome:**				
Mean Bayley III Scale of Infant and Toddler Development Scaled Scores				
Cognitive Scale b	7.8 (1.8)	7.2 (2.4)	111	2.45, 0.12
Communication Scale b	14.6 (5.0)	15.2 (4.1)	111	0.37, 0.54
Expressive Communication b	8.3 (2.4)	8.7 (2.3)	111	0.82, 0.37
Receptive Communication b	6.7 (2.5)	6.7 (1.9)	111	0.01, 0.94
Motor Scale b	17.8 (4.2)	18.9 (4.4)	112	1.78, 0.19
Fine Motor b	9.2 (3.0)	8.9 (2.2)	112	0.30, 0.58
Gross Motor b	8.6 (3.0)	10.0 (3.2)	112	5.35, 0.02*
Social and Emotional Scale b	10.5 (3.2)	10.2 (3.2)	111	0.20, 0.66
Adaptive Behaviour Scale b	78.8 (17.8)	81.6 (13.9)	111	0.79, 0.38
**Secondary Outcomes:**				
Edinburgh Postnatal Depression Score				
N (%) ≥13	5 (8.1)	3 (5.8)	112	.25, .45
Mean (sd)	6.8 (4.9)	5.6 (4.6)	112	1.38, 0.24
Median (IQR, range)	6 (7, 0–26)	5 (5, 0–24)		0.12a
Mother to Infant Bonding Score				
Mean (sd)	1.4 (2.8)	1.0 (1.8)	111	0.30, 0.59
Median (IQR, range)	0 (2, 16–28)	0 (1, 16–28)		0.95 a
Pearlin Self efficacy Scale				
Mean (sd)	23.1 (3.4)	23.7 (3.5)	111	0.83, 0.37
Median (IQR, range)	23 (6, 0–13)	24 (6, 0–8)		0.20 a
Proportion of Mind-minded Comments				
Mean (sd)	25.7 (22.0)	27.0 (20.6)	111	0.17, 0.68
Median (IQR, range)	20.00 (39.28, 0–75)	25 (27.50, 0–75)		0.45a
Mean Baseline to Follow up change group differences				
Edinburgh Postnatal Depression Score (sd)	0.46 (5.25)	0.64 (5.08)	111	0.75, 0.39
Mother to Infant Bonding Score (sd)	-0.09 (3.13)	0.06 (2.03)	107c	0.10, 0.75
Pearlin Mastery Scale (self efficacy) (sd)	3.58 (3.53)	3.22 (3.36)	109d	1.72, 0.19

*p ≤ 0.05.

Main analyses use ANCOVA with gestation in days as covariate (gestation information missing n = 1), except: a Mann Whitney Test used to analyse group differences; b ANOVA used as Bayley scaled scores are calculated using infant adjusted age. c missing mother to infant bonding data at baseline (n = 5); d missing self efficacy data at baseline (n = 3). Baseline measures taken at 8–12 weeks postpartum.

## Discussion

This study aimed to examine whether the POW service had a positive impact on infant development at 12 months and a sustained effect on maternal depression, self efficacy and bonding at 12 months postpartum. There was no evidence of sustained improvements in any aspect of maternal mental health in the sub-sample who completed follow up but infants of women who received the POW intervention had significantly better gross motor skills at 1 year than infants of women who received standard care. There were no other significant improvements in any other aspect of development in infants in the POW arm of the trial.

Gross motor skills include the assessment of general musculature, physical skills and coordination, and thus gross motor skills delay is often used as an indicator of developmental delay. The infants of women who received the POW support demonstrated gross motor skills in line with that expected of their age group using scaled reference scores. In contrast, their peers who did not receive the intervention demonstrated gross motor skill performance significantly below the norm for their age, consistent with their background of high social risk. In general, the infants performed less well than a same aged peer reference group, particularly in indices of cognitive and receptive communication development, for which they scored over one standard deviation below that expected of their age group (between the 9th and 16th percentile).

Delayed development of motor skills has been associated with later cognitive deficits such as dyslexia, ADHD and language impairment [48]. Specifically, gross motor skills (but not fine motor skills) measured from birth to 4 years have been shown to explain significant variance in children’s later cognitive performance, particularly working memory and processing speed [49]. Therefore, whilst we did not demonstrate a significant effect of the intervention on cognitive development at 12 months, it is possible that children whose mothers received the POW intervention may show better longer term cognitive outcomes. This may be particularly important in this group, who are showing signs of significant underperformance in many areas of development.

The positive effects of the POW service on postnatal mental health are likely to have benefitted maternal-infant interaction, given that self efficacy is associated with greater parental competence [35] and that maternal depression is associated with less play, touch and affection [12]. Therefore, one mechanism by which the POW service had positive effects on infant gross motor outcome may be through better sensorimotor stimulation. Another mechanism may be that of exposure to a less stressful uterine environment. Maternal antenatal distress has previously been associated with poorer motor development in infancy [50] and is a more important predictor of motor development than postnatal distress. Aspects of distress clustered in the second and third trimesters, including anger, pregnancy related stress and cortisol levels, have specific links to poorer infant psychomotor development [51]. There are a number of other potential mechanisms linking improvements in early postnatal mental health to better later infant gross motor outcomes, include greater support from a less depressed mother to infant attempts at movement, as well as general improvements in environmental stimulation (toys, opportunities) that increase children’s interest in their environment and motivate the development of movement and action. Furthermore, improvements in bonding from mother to infant facilitate security of attachment and thus may improve willingness to explore the environment. Improvements in maternal pregnancy health (for example reductions in the use of drugs and/or alcohol during pregnancy, reduced use of antidepressants during pregnancy) may also have implications for improvements in infant gross motor development. However, we do not have data from the ELSIPS study to enable us to examine these factors as mechanisms, so these conclusions remain speculative.

One important limitation of this study was that the sample selected for follow up was pragmatic, limited by financial and time restrictions, and a less diverse, less depressed and more self- efficacious group were tested for longer term outcomes. Selection bias regarding inclusion into the follow up study applied equally to both arms of the study but unobserved selection mechanisms may have comprised the comparability of the study groups despite the efforts of the investigators to apply selection criteria in both arms in a symmetrical way. Thus the lack of representativeness of our follow up sample does pose significant limitations on our ability to make conclusions about the effectiveness of the overall ELSIPS trial on later infant outcome. Also, whilst there was a significant difference in self efficacy between the follow up sample POW and standard care participants at 8–12 weeks, the differences in depression at that time were slight. Therefore, it is possible that the effects of the intervention on maternal mental health are relatively short term or that the measured effects of the intervention on long term maternal mental health, bonding and infant development may be underestimated. A further important limitation of the study is that of multiple testing. We did not undertake Bonferroni correction for multiple testing given their unnecessarily stringent effects because we did not want to increase the risk of type II error in this relatively small sample. However, this raises the possibility that the only improvement in infant development found in this study, that of gross motor development, could be a spurious finding resulting from a type I error. Nevertheless the scale of the effect (half a standard deviation difference between arms) suggests that this result is a clinically significant one. Further work examining the effects of pre- and post-natal lay interventions on longer term infant outcomes, and the precise mechanisms of action, is required.

There is a need for a greater range of support services for perinatal women with social risks, including support in addressing economic and social needs, and these women express a preference for services focused on empowerment of the women themselves [52]. The POW service is an example of such a service, which shows positive effects on maternal mental health and bonding in the short-term. Whilst the benefits of the POW service for infant development are limited, there may be a benefit for infant gross motor outcomes in the longer term.

## Supporting information

S1 Table ELSIPS follow up sample composition and characteristics in mothers with two or more social risks only. (DOCX)

S2 Table Group differences in mothers with two or more social risks in the ELSIPS follow up study at 12 months postpartum. (DOCX)

## Abbreviations

ELSIPS: Evaluation of Lay Support in Pregnant women with Social risk
