# Supplementary material for: Effects of lay support for pregnant women with social risk factors on infant development and maternal psychological health at 12 months postpartum
Source: PLoS One. 2017 Aug 28;12(8):e0182544. doi: 10.1371/journal.pone.0182544 (PMC5573293; doi:10.1371/journal.pone.0182544)
Supplement: S1 Table — (DOCX) [file pone.0182544.s001.docx]

**Supporting Information**

Table S1. ELSIPS Follow Up Sample Composition and Characteristics in Mothers with Two or More Social Risks Only

|  | **Treatment Group** | | |
| --- | --- | --- | --- |
|  | **standard** | **POW** | **total** |
| ELSIPS sample |  |  | 1324 |
| Eligible to participate |  |  | 486 |
| Contacted by researcher |  |  | 155 |
| Recruited (%) |  |  | 112 |
| Social Risk Count of 2 or More | 43 (50) | 43 (50) | 86 |
| Boy (%) | 27 (62.8) | 24 (55.8) |  |
| Girl (%) | 16 (37.2) | 19 (44.2) |  |
| Ethnicity |  |  |  |
| White European (%) | 33 (76.7) | 30 (69.8) |  |
| African (%) | 1 (2.3) | 4 (9.3) |  |
| Caribbean (%) | 5 (11.6) | 4 (9.3) |  |
| Asian (%) | 2 (4.7) | 1 (2.3) |  |
| Other (%) | 2 (4.7) | 4 (9.3) |  |
| Birthweight (sd) | 3169.5 (597.3) | 3057 (717) |  |
| Gestation in days (sd) | -0.6 (10.4) | -5.9 (17)* |  |
| Social risk count (sd) | 2.2 (1.1) | 2.5 (0.9)* |  |

*p<.05.
