# Supplementary material for: Effects of lay support for pregnant women with social risk factors on infant development and maternal psychological health at 12 months postpartum
Source: PLoS One. 2017 Aug 28;12(8):e0182544. doi: 10.1371/journal.pone.0182544 (PMC5573293; doi:10.1371/journal.pone.0182544)
Supplement: S2 Table — (DOCX) [file pone.0182544.s002.docx]

**Supporting Information**

Table S2. Group Differences in Mothers with Two or More Social Risks in the ELSIPS Follow up Study at 12 months postpartum

|  | Treatment Group | | | | |  |
| --- | --- | --- | --- | --- | --- | --- |
|  | Standard | | POW | |  |  |
| Outcome variable | Mean | sd | Mean | sd | N | F, *p-value* |
| Primary: Bayley III Scale of Infant and Toddler Development |  |  |  |  |  |  |
| Cognitive Scale^a^ | 7.7 | 2.0 | 7.2 | 2.4 | 85 | 1.13, 0.29 |
| Communication Scale^a^ | 14.5 | 4.9 | 18.9 | 25.9 | 85 | 1.21, 0.28 |
| Expressive Communication^a^ | 8.3 | 2.6 | 8.6 | 2.3 | 85 | 0.26, 0.61 |
| Receptive Communication^a^ | 6.4 | 2.4 | 6.5 | 1.8 | 85 | 0.11, 0.75 |
| Motor Scale^a^ | 18.1 | 4.4 | 19.0 | 4.6 | 86 | 0.83, 0.36 |
| Fine Motor^a^ | 9.3 | 3.2 | 8.9 | 2.1 | 86 | 0.45, 0.51 |
| Gross Motor^a^ | 8.7 | 3.1 | 10.0 | 3.3 | 86 | 3.38, 0.07 |
| Social and Emotional Scale^a^ | 10.4 | 2.8 | 10.3 | 3.1 | 85 | 0.02, 0.89 |
| Adaptive Behaviour Scale^a^ | 80.7 | 18.2 | 81.6 | 14.9 | 85 | 0.06, 0.82 |
| Secondary Outcomes: |  |  |  |  |  |  |
| Edinburgh Postnatal Depression Score | 7.7 | 5.3 | 5.7 | 4.8 | 86 | 2.17, 0.15 |
| Mother to Infant Bonding Score | 1.6 | 2.8 | 0.9 | 1.9 | 86 | 1.00, 0.32 |
| Pearlin Mastery Scale (self efficacy) | 22.6 | 3.5 | 23.6 | 3.6 | 86 | 1.40, 0.24 |
| Proportion of Mind-minded Comments | 25.0 | 20.5 | 26.3 | 20.8 | 86 | 0.26, 0.62 |
|  |  |  |  |  |  |  |
| Baseline to Follow up change group differences |  |  |  |  |  |  |
| Edinburgh Postnatal Depression Score | 1.0 | 5.6 | 0.7 | 5.2 | 86 | 0.02, 0.90 |
| Mother to Infant Bonding Score | -0.1 | 2.4 | 0.1 | 2.1 | 82 | 0.26, 0.61 |
| Pearlin Mastery Scale (self efficacy) | 3.4 | 3.3 | 3.3 | 3.4 | 85 | 0.06, 0.80 |
